# International Collaboration and Commercial Involvement in Randomized Controlled Trials From 10 Leading Countries, 1997 Through 2019

**DOI:** 10.7759/cureus.61205

**Published:** 2024-05-27

**Authors:** Shunichi Fukuhara, Yuki Kataoka, Takuya Aoki, Joseph Green, Sayaka Shimizu, Nagayasu Toyoda

**Affiliations:** 1 Section of Clinical Epidemiology, Department of Community Medicine, Graduate School of Medicine, Kyoto University, Kyoto, JPN; 2 Department of Health Policy and Management, Johns Hopkins Bloomberg School of Public Health, Baltimore, USA; 3 Department of General Medicine, Shirakawa Satellite for Teaching And Research (STAR) and Center for Innovative Research for Community & Clinical Experience (CiRCLE) Fukushima Medical University, Fukushima, JPN; 4 Department of Internal Medicine, Kyoto Min-iren Asukai Hospital, Kyoto, JPN; 5 Division of Clinical Epidemiology, Research Center for Medical Sciences, The Jikei University School of Medicine, Tokyo, JPN; 6 Graduate School of Medicine, The University of Tokyo, Tokyo, JPN; 7 Department of Research, Patient Driven Academic League (PeDAL), Tokyo, JPN; 8 Department of Obstetrics and Gynecology, Suzuka University of Medical Science, Suzuka, JPN

**Keywords:** core clinical journals, medical publication, commercial involvement, international collaboration, randomized controlled trials

## Abstract

Randomized controlled trials (RCTs) affect clinical decisions and their number is increasing. However, trends in international collaboration on RCTs and involvement of healthcare-related industries, the latter of which may contribute to bias, are not known. The objectives were to identify concerns surrounding RCTs, and to quantify changes in (1) the numbers of RCT articles in journals of high clinical importance, (2) international collaboration, and (3) commercial involvement in RCTs by authors in countries that contribute the most to the scientific literature. This was not a systematic review of the medical literature. It is a descriptive study of trends during the past two decades. We extracted RCT articles from MEDLINE data (1997-2019). When grouped by authors' country, the analyses were limited to the 10 leading countries in the natural sciences, as defined by the Nature Index 2019 Annual Tables. The Core Clinical Journals (CCJ) filter in PubMed was used to identify journals that were likely to be highly relevant to clinical practice. RCT articles that included authors from multiple countries were used as examples of international collaboration, and RCTs in which at least one author’s affiliation was corporate were considered to have commercial involvement. The annual number of RCT articles more than doubled (from 10,360 to 22,384), but the number published in the CCJ was essentially unchanged (from 2,245 to 2,346). The vast majority of RCT articles had US-based authors. International collaboration increased in nine of the 10 countries studied, and it was particularly common among researchers in Europe, Canada, and Australia. In contrast, international collaboration decreased in China. Regarding commercial involvement, between 1997 and 2019 the proportion of single-country RCTs with commercial involvement decreased (from 12.4% to 3.8% for the United States, and from 2.5% to 0.0% for Europe-Canada-Australia). In contrast, the proportion of international-collaborative RCTs with commercial involvement increased (from 9.2% to 17.6% for the United States, and from 17.9% to 21.3% for Europe-Canada-Australia). The largest change in commercial involvement was the 12-fold increase in Japan: from 3% to 36% (1997-2019). Japan was also noteworthy for its 28-percentage-point decrease in first-authorship of RCT articles from 2012 to 2019. In conclusion, recent increases in the number of RCT articles have occurred almost exclusively outside the CCJ. Thus, many newer RCT articles might have relatively low clinical relevance or impact. International collaboration has generally increased, along with commercial involvement. The latter has become particularly common in Japan, increasing the potential for sponsorship bias. The effects of ongoing attempts to reverse that trend should be evaluated.

## Introduction and background

Evidence from randomized controlled trials (RCTs) informs clinical decisions. We are concerned here with three trends in RCTs over recent decades. First, the number of RCT articles has increased. For example, it increased from 9,486 in 1995 to 22,560 in 2017 in the journals indexed in PubMed/MEDLINE, the largest and most comprehensive database of the medical literature [1,2]. Second, not only has the Internet made scientific communication faster and simpler than before, but also the number of face-to-face contacts at meetings of international associations in all areas of bio-medical research has also grown, and international travel has become more affordable, which facilitates exchanges of faculty, students, and clinical-research trainees. Those increases in international connections within academic medicine might have affected international-collaborative RCTs. Third, trends in international collaboration may have been related to trends in commercial involvement with RCTs (e.g., funding of RCTs by pharmaceutical and medical device companies, and co-authorship of RCT articles by employees of those companies).

Those three trends can have clinical consequences. While the number of RCT articles indexed in PubMed/MEDLINE has increased, some RCT articles may have more impact on clinical decisions than others. Our primary concern was with those RCT articles that are most likely to have the greatest effect on clinical decisions. One possible proxy for the clinical impact or relevance of an RCT article is its publication in one of the Core Clinical Journals (CCJ). The CCJ list was created by the U.S. National Library of Medicine [3]. Journals among the CCJ are likely to be highly relevant to clinical practice. The CCJ includes 118 journals and its PubMed filter was used to identify journals for inclusion in this study. Commercial involvement in RCTs is likely to be associated with distortion of study design and reporting to favor the sponsor, which is a phenomenon known as sponsorship bias [4,5]. We focused in part on international-collaborative RCTs, because, in contrast to sponsorship bias in single-country RCTs, sponsorship bias in international-collaborative RCTs may negatively affect more decisions by more clinicians worldwide, because the results of those studies will be assumed to be valid in many countries. Sponsorship bias has been recognized for more than 100 years [6]. Specifically, the research questions of greatest commercial interest may differ from the questions that are most relevant to patients and their families, to clinicians, and to society. This leads to concern that involvement in commercial activities might redirect researchers’ time away from clinically important research questions. Furthermore, even without affecting research priorities, and despite many years of attention to these issues, commercial involvement in clinical research is still associated with biased results that can adversely affect clinical decisions [7,8]. With those concerns in mind, we sought to clarify the recent past and the present situation of RCTs, particularly highlighting (a) the numbers of articles indexed in MEDLINE (whether they were published in the CCJ or elsewhere), (b) international collaboration, and (c) commercial involvement. We focused on RCTs by authors from the 10 leading countries in the natural sciences (as defined below), because of the inherent obstacles to analyses and interpretations of annual trends in data from countries with few annual RCT publications (obstacles that are referred to again in the Materials & Methods and Discussion sections below).

## Review

Materials & methods

Data Sources

Publications of RCTs’ results indexed in MEDLINE were identified by searching PubMed [9]. We used MEDLINE not merely because it is among the largest databases of the worldwide bio-medical research literature [2]. MEDLINE was appropriate for this study, because, rather than conducting a comprehensive search across multiple databases, we sought to longitudinally examine RCTs published in the CCJ. The reason is that RCTs published in the CCJ are likely to be highly relevant to clinical practice. MEDLINE was chosen as the database for this study, as it comprehensively indexes the CCJ over the time period of interest. The search date was 12 April, 2021 and thus the search was done with the CCJ filter that was in effect on that date. Using the code in the Appendix, we searched for RCT articles published from 1997 to 2019 because there is a one-year delay in the assignment of search tags in MEDLINE.

Eligibility Criteria

We limited the search to RCT articles published from 1997 through 2019, without regard to the type of intervention or medical condition studied. The analyses grouped by authors' country were limited to the “top 10 countries that dominate natural-sciences research” published in the Nature Index 2019 Annual Tables [10]: Australia, Canada, China, France, Germany, Japan, South Korea, Switzerland, the United Kingdom, and the United States (in alphabetical order). This ranking was based on a country’s publication output in 82 natural science journals, selected by an independent panel of scientists who were leaders in their fields. It was necessary to limit this study to the top-ranked countries only. The reason is that, unfortunately, if a country has few annual RCT publications then the annual data from that country are less amenable to meaningful analyses, with the consequence that, for practical purposes, useful comparisons and interpretations are not possible. This is discussed in more detail in the Limitations section.

For some analyses, the data on Germany, the United Kingdom, France, Switzerland, Canada, and Australia were combined into one category (abbreviated as “Europe-Canada-Australia”). That category was used because it clearly illustrates how international collaboration can overcome the problem of doing clinically useful RCTs when local populations are relatively small. While both Japan and South Korea are much smaller than the US and China, here they were considered separately. That was done mainly because of their relatively unique recent history of changes in the publication of RCT articles.

Assignment of Country Affiliation

We counted the number of authors from PubMed's Author (AU) and Investigator Name (IR) fields. After March 2008, the IR field was used to contain personal names of individuals who are not authors of a paper (e.g., collaborators and investigators) but are listed in the paper as members of a collective/corporate group that is an author of the paper in MEDLINE [11]. The country affiliation of each first author was that author’s country and/or place name in PubMed. If an author was affiliated with more than one country, that author’s affiliation was considered to be the first country listed on PubMed. Country affiliations of all authors were determined using data from InCites. Based on the Web of Science, InCites is a tool that includes information on countries of each institutional affiliation that may not be included in other databases [12].

Statistical Analysis

Descriptive statistics showing trends over time were computed using the following methods.

We described the annual changes in the number of RCT articles and the number of journals publishing RCT articles. In addition, limited to RCT articles from the CCJ, the annual trend in the number of RCT articles and the median number of authors per RCT article are shown. To focus on the RCTs that are most likely to affect clinical practice, the following analyses were conducted with articles from the CCJ.

To quantify each country’s (or category’s) share of RCTs published in the CCJ, we counted the number of RCT articles in which at least one author was affiliated with that country (or category). This allowed each article to contribute to the total count for more than one country.

Using each country’s data from each year, we computed an index of international collaboration. It was a proportion, expressed below as a percentage. Its denominator for each country was, among all RCT articles published in the CCJ, the number of those articles that had at least one author from that country. For example, the denominator for the US was, among all RCT articles published in the CCJ, the number of those articles that had at least one author from the US. This was the number of US-co-authored RCT articles in the CCJ. For those proportions, the numerator for each country was, among all of the RCT articles in the denominator for that country, the number of those articles that had at least one author from a different country. Thus, the lowest and highest possible values are 0 and 1 (0% and 100%), and higher values reflect more international collaboration.

To examine commercial involvement in RCTs, we calculated the proportion of reports of such RCTs in the CCJ each year from 1997 to 2019 for each country. Commercially-involved RCT articles were those in which one or more of the co-authors’ affiliations were corporate. For each country, the denominator was the number of RCT articles in the CCJ (any author) each year, and the numerator was the number of commercially-involved RCT articles in the CCJ (any author). Next, distinguishing articles reporting commercially involved single-country RCTs from those reporting commercially involved international-collaborative RCTs, we computed the proportions of those two types separately.

To examine trends in the first author’s country, we calculated the proportion of the first authored RCT articles in the CCJ by country or category. The denominator was the number of RCT articles in the CCJ for that country or category. The numerator was the number of RCT articles on which the first author’s affiliation was that country or category.

We used Python v.3.6.4 (Python Software Foundation, Wilmington, DE, USA) with the Biopython library v.1.73 to search PubMed and to compute summary statistics [13]. We adopted complete case analysis when calculating proportions, i.e., we excluded MEDLINE records that were not indexed in InCites.

Results

**Figure 1:**
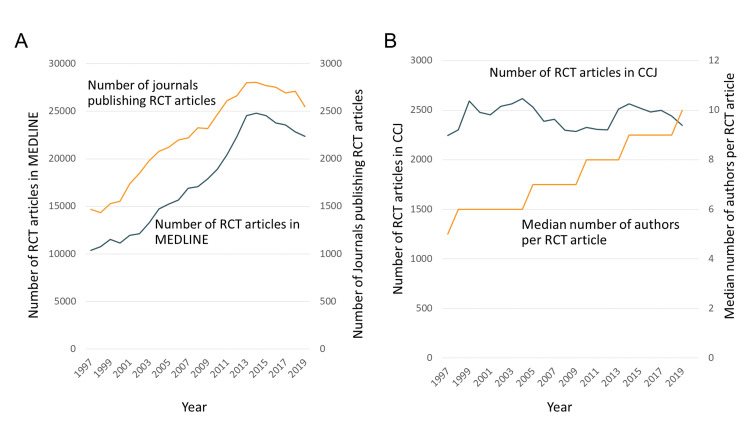
Number of RCT articles, 1997 through 2019 A. Number of RCT articles in MEDLINE, and number of journals B. Number of RCT articles, and number of authors of RCTs in the CCJ Data were from MEDLINE via PubMed search. RCT: Randomized Controlled Trial; CCJ: Core Clinical Journals.

The number of RCT articles in all journals increased, from 10,360 in 1997 to 22,384 in 2019. Over the same time, the number of journals in which RCTs were published increased by 73%, from 1,470 to 2,547 (Figure 1A). In contrast, when the view was restricted to the CCJ, the number of RCT articles published annually was largely unchanged over 22 years (from 2,245 in 1997 to 2,346 in 2019) (Figure 1B).

**Figure 2:**
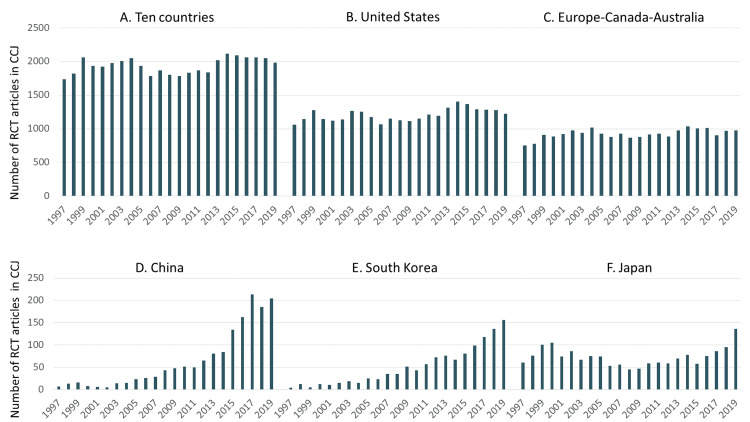
Number of RCT articles in the CCJ, by country or category The ten countries were Australia, Canada, China, France, Germany, Japan, South Korea, Switzerland, the United Kingdom, and the United States (in alphabetical order). Note the difference in vertical scales between the three upper panels and the three lower panels. Data were from MEDLINE via PubMed search. RCT: Randomized Controlled Trial; CCJ: Core Clinical Journals.

As shown in Figure 2A-2F, US-based authors were included in more RCT articles than authors based in Europe-Canada-Australia. For most of the time period studied, US-based authors were also included in approximately 10 times as many RCT articles as were authors based in China, South Korea, or Japan.

**Figure 3:**
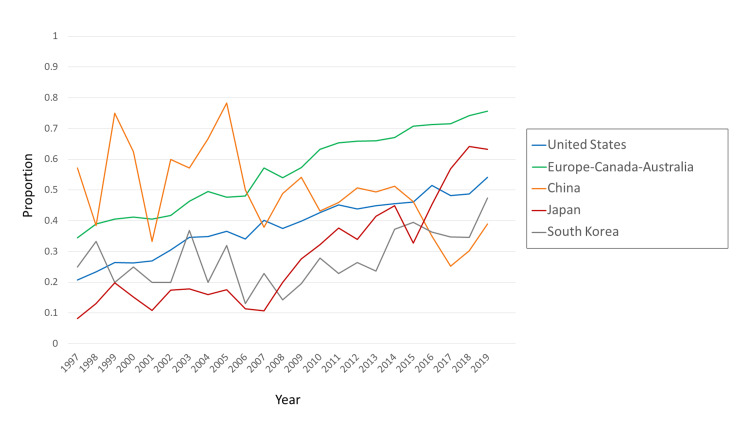
Proportion of international-collaborative RCT articles in the CCJ, by country or category The denominator was the number of RCT articles in the CCJ, for each country or category. The numerator was the number of RCT articles with at least two different countries in the address field, for each country or category. Data were from MEDLINE via PubMed search and InCites. RCT: Randomized Controlled Trial; CCJ: Core Clinical Journals.

Trends in international collaboration differed by country (Figure 3). For researchers based in Europe-Canada-Australia, international collaborations were consistently more common than they were for US-based researchers, although both increased steadily over 22 years.

Among the Asian countries studied, the index of international collaboration varied widely over time and between countries. South Korean involvement in international-collaborative RCTs increased greatly between 2008 and 2019 (Figure 3). For Japan, international collaboration increased slowly between 1997 and 2004, but between 2015 and 2019 it increased quickly and substantially, by 30 percentage points from 33% to 63% (Figure 3). For China-based researchers, the changes in the index of international collaboration over 22 years were unlike those of any other country (or category). However, the wide fluctuations in that index from 1997 to 2007 (Figure 3) are probably not very meaningful, because they are large changes in a proportion based on very small absolute numbers (<50/year, Figure 2D). Between 2012 and 2019, when the absolute numbers were somewhat higher (>50/year, Figure 2D), the index of international collaboration decreased by 12 percentage points, from 51% to 39% (Figure 3).

**Figure 4:**
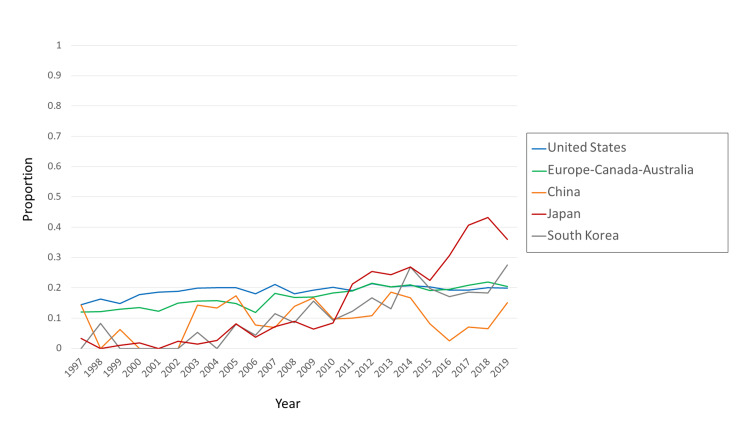
Proportion of commercially involved RCT articles in the CCJ, by country or category Commercially involved RCT articles were those in which one or more of the co-authors’ affiliations were “corporate”. The denominator was the number of RCT articles in the CCJ, for each country or category. Data were from MEDLINE via PubMed search and InCites. RCT: Randomized Controlled Trial; CCJ: Core Clinical Journals.

Commercial involvement varied by country and over time (Figure 4). The large fluctuations in China’s proportions before approximately 2012 are difficult to interpret, for the reason mentioned above. Most notable is the 12-fold increase in Japan (from 3% to 36% between 1997 and 2019).

**Figure 5:**
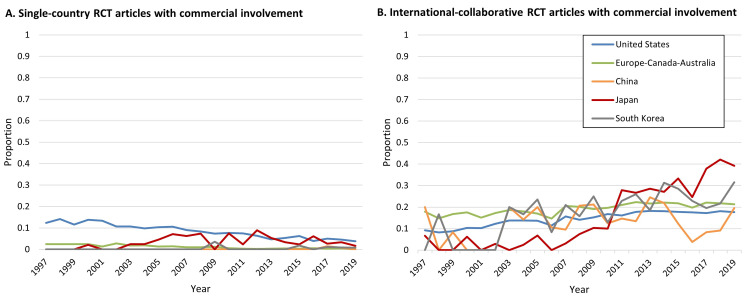
Proportion of commercially involved RCT articles in the CCJ, by type of RCT (single-country or international-collaborative), and by country or category A. Proportion of single-country RCT articles in the CCJ. The numerator was the number of single-country RCT articles with commercial involvement, for each country or category. B. Proportion of international-collaborative RCT articles. The numerator was the number of RCT articles that had at least two different countries in the address field and also had commercial involvement for each country or category. In both A and B, the denominator was the number of RCT articles in the CCJ, for each country or category. Commercially involved RCT articles were those in which one or more of the co-authors’ affiliations were “corporate”. Data were from MEDLINE via PubMed search and InCites. RCT: Randomized Controlled Trial; CCJ: Core Clinical Journals.

Commercial involvement also varied by the type of RCT (single-country vs international-collaborative), by country, and over time, as follows. When single-country RCTs and international-collaborative RCTs were analyzed separately, the index of commercial involvement was unstable ("noisy") for some countries, apparently because of low numbers of observations, whereas for the United States and Europe-Canada-Australia, it was stable enough to be interpretable. Between 1997 and 2019 the proportion of single-country RCTs with commercial involvement decreased (from 12.4% to 3.8% for the United States, and from 2.5% to 0.0% for Europe-Canada-Australia, Figure 5A). In contrast, the proportion of international-collaborative RCTs with commercial involvement increased (from 9.2% to 17.6% for the United States and from 17.9% to 21.3% for Europe-Canada-Australia, Figure 5B).

**Figure 6:**
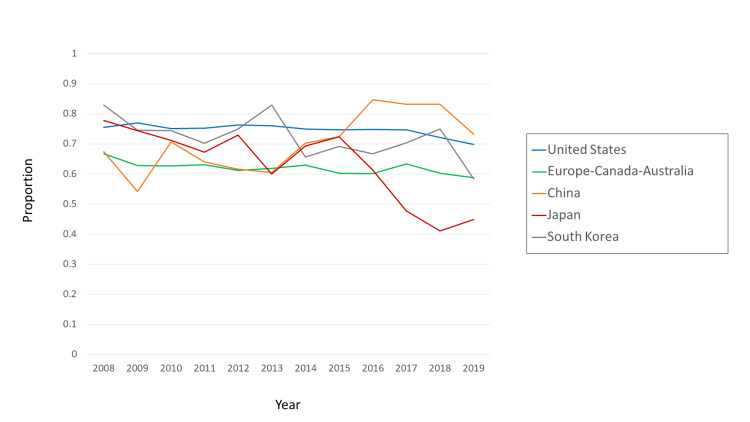
Proportion of first-authored RCT articles in the CCJ, by country or category The denominator was the number of RCT articles in the CCJ, for that country or category. The numerator was the number of RCT articles in which the first author’s affiliation was that country or category. Data were from MEDLINE via PubMed search and InCites. RCT: Randomized Controlled Trial; CCJ: Core Clinical Journals.

One other difference between China and Japan is noteworthy. From 2012 through 2019, first-authorship by China-based researchers increased by about 12 percentage points, while first-authorship by Japan-based researchers decreased by 28 percentage points (Figure 6).

Discussion

From 1997 through 2019, the number of RCT articles published in MEDLINE-indexed journals more than doubled. Nonetheless, the number of RCT articles published in the CCJ was rather stable (Figure 1B). Therefore, that increase in RCT articles was almost completely confined to journals outside the CCJ, possibly including newer, online-only journals that can easily publish a large number of articles. If the publication of an RCT article in one of the CCJ can be used as an indicator that the underlying RCT is of relatively high clinical relevance or impact, then Figure 1B would show that the contribution of newer, online-only journals to the medical literature has been to increase the number of RCTs, while their influence on clinical decisions remains in doubt.

In Asian countries, RCT-article production was at extremely low levels in 1997, after which it increased gradually over the next two decades. Nonetheless, consistently during that period, the numbers from the US far exceeded those from China, South Korea, or Japan.

With all 10 countries considered together, the index of international collaboration more than doubled between 1997 and 2019: from 20% to 47%. International collaborations were particularly common for researchers based in Europe-Canada-Australia (Figure 3), comprising more than 75% of the RCTs from those countries in 2019. During the period under study, the difference between the US and Europe-Canada-Australia increased from 14 percentage points to 22 percentage points. The difference between China and Europe-Canada-Australia was even greater: 37 percentage points in 2019. Researchers based in Europe-Canada-Australia might have an incentive for collaboration that is not shared by those based in the US or in China: statistical power. Specifically, international collaboration may provide the large sample sizes needed for some RCTs. Another reason for increases in international collaboration might be that the global reach of pharmaceutical and medical device companies has increased the benefits of conducting international NDA-oriented trials (through successful harmonization by ICH [14]) and post-marketing trials. Any increases in the number of international NDA-oriented trials and international post-marketing trials could raise the likelihood of accompanying increases in the number of cases of sponsorship bias, which would, of course, be of great concern to clinicians worldwide. The finding that between 1997 and 2019 the proportion of single-country RCTs with commercial involvement decreased while the proportion of international-collaborative RCTs with commercial involvement increased (Figure 5) shows the necessity of attention to international-collaborative RCTs as part of efforts to carefully monitor commercial involvement and the potential for sponsorship bias.

With regard to China, the number of RCT articles increased slowly from 1997 to 2014, and then it increased rapidly (Figure 2D). After about 2014, the number of RCT articles increased, but the index of international collaboration decreased (Figure 3). Thus, while emerging onto the world stage with regard to the production of RCTs, China also appeared to become less interdependent with other countries, taking a path that was unique among the countries studied. Analyzing possible causes of that trend is far beyond the scope of the present study.

The prevalence of commercial involvement in RCTs appeared to be rather stable over 22 years (11% in 1997 and 13% in 2019) when all 10 countries were considered together. However, that apparent stability conceals large differences over time and across countries. As of 2019, commercial involvement was particularly important in Japan (36%), while playing a far smaller role in China (15%), and Japan and China had been moving in opposite directions on this metric since about 2015 (Figure 4). We note that Japan and China also moved in opposite directions regarding first authorship (Figure 6).

The situation of Japan is particularly disturbing. The combination of a large increase in commercial involvement and a large decrease in first authorship was not seen in any of the other countries studied. Japan’s extreme reliance on industry sponsorship of RCTs should also be considered in a historical context. In 2012, a highly-publicized scandal erupted in Japan, involving an industry-sponsored RCT of Valsartan [15]. Despite the fact that the scandal resulted in public condemnation of the pharmaceutical industry, industry-sponsored RCTs nonetheless resumed their growth in Japan. In 2018, they reached 43%, which is the highest percentage of industry sponsorship in the 10 countries in this study for any of the 22 years examined. It is also more than twice as high as those of South Korea and the US. Recently in Japan, a new law was passed on clinical investigations. That new law extends the regulation of NDA-oriented RCTs to include non-NDA RCTs sponsored by industry [16]. If that new law causes industry funding of investigator-initiated post-marketing studies to decrease, then the total number of RCTs done in Japan might also decrease, as there are few other sources of funding for those studies. In 2015, the Japan Agency for Medical Research and Development (AMED [17]) was established to provide publicly-funded support for basic and clinical research. Since that extension of the regulations came into effect only in 2017, and because AMED is still young, their long-term effects on RCTs and sponsorship bias in Japan remain to be seen. South Korea might be an example of how a relatively small country can increase its production of RCTs without extreme dependence on industry sponsorship.

Limitations

Here we note some limitations of this study. First, the ranking of countries published in Nature in 2019 applies not only to research that informs clinical medicine but also to research in all natural sciences. Also, some RCT articles published outside the CCJ probably affect clinical practice. To the best of our knowledge, the reliability and validity of RCT results published in the CCJ have not been compared with those published in other journals, and the present findings do not incorporate an index of RCTs’ quality. In addition, the number of authors before 2008 is truncated, and the search filter we used to identify RCTs is very specific but is only 83% sensitive [18]. We believe it is nonetheless useful for documenting general trends. A more sensitive and specific operational definition of commercial involvement in RCTs could also be useful. Finally, considering Germany, the United Kingdom, France, Switzerland, Canada, and Australia as one group could have resulted in some loss of information. However, the absolute numbers of RCTs from those countries were low, i.e., the data were sparse. Therefore, the percentage-based indices (international collaboration and commercial involvement) for each of those countries would have been extremely sensitive to outliers and noise in the data. Thus, if those six countries had been considered individually, their indices of international collaboration and commercial involvement would have been uninterpretable. Considering those countries individually might be useful in future studies, but only if the numbers of RCT articles are sufficiently large, or if analytic methods for minimizing sparse-data bias are applicable.

## Conclusions

Recent increases in the number of RCT articles have occurred almost exclusively outside the CCJ. Thus, many newer RCT articles might have relatively low clinical relevance or impact. International collaboration has generally increased, along with commercial involvement. The latter has become particularly common in Japan, increasing the potential for sponsorship bias. The effects of ongoing attempts to reverse that trend should be evaluated.

## Appendices

**Table 1:** Code for the search According to the journal’s submission rules, “[” is replaced with “|” and “]” with “!” in the code.

Code
import os
import pandas as pd
from pandas import DataFrame
import math
from Bio import Entrez
from Bio import Medline
import time
import numpy as np
#%%
term = '1997|pdat!:2999|pdat! AND Randomized Controlled Trial|publication type!'
ccj = '"Academic medicine : journal of the Association of American Medical Colleges"|journal! OR "AJR. American journal of roentgenology"|journal! OR "American family physician"|journal! OR "American heart journal"|journal! OR "The American journal of cardiology"|journal! OR "The American journal of clinical nutrition"|journal! OR "American journal of clinical pathology"|journal! OR "The American journal of medicine"|journal! OR "The American journal of nursing"|journal! OR "American journal of obstetrics and gynecology"|journal! OR "American journal of ophthalmology"|journal! OR "the American journal of pathology"|journal! OR "American journal of physical medicine & rehabilitation"|journal! OR "The American journal of psychiatry"|journal! OR "American journal of public health"|journal! OR "American journal of respiratory and critical care medicine"|journal! OR "American journal of surgery"|journal! OR "The American journal of the medical sciences"|journal! OR "The American journal of tropical medicine and hygiene"|journal! OR "Anaesthesia"|journal! OR "Anesthesia and analgesia"|journal! OR "Anesthesiology"|journal! OR "Annals of emergency medicine"|journal! OR "Annals of internal medicine"|journal! OR "The Annals of otology, rhinology, and laryngology"|journal! OR "Annals of surgery"|journal! OR "The Annals of thoracic surgery"|journal! OR "Archives of disease in childhood"|journal! OR "Archives of disease in childhood. Fetal and neonatal edition"|journal! OR "Archives of environmental & occupational health"|journal! OR "Archives of pathology & laboratory medicine"|journal! OR "Archives of physical medicine and rehabilitation"|journal! OR "Arthritis & rheumatology (Hoboken, N.J.)"|journal! OR "BJOG : an international journal of obstetrics and gynaecology"|journal! OR "Blood"|journal! OR "BMJ (Clinical research ed.)"|journal! OR "The bone & joint journal"|journal! OR "Brain: a journal of neurology"|journal! OR "The British journal of radiology"|journal! OR "The British journal of surgery"|journal! OR "CA: a cancer journal for clinicians"|journal! OR "Cancer"|journal! OR "Chest"|journal! OR "Circulation"|journal! OR "Clinical orthopaedics and related research"|journal! OR "Clinical pediatrics"|journal! OR "Clinical pharmacology and therapeutics"|journal! OR "Clinical toxicology (Philadelphia, Pa.)"|journal! OR "CMAJ"|journal! OR "Critical care medicine"|journal! OR "Current problems in surgery"|journal! OR "Diabetes"|journal! OR "Digestive diseases and sciences"|journal! OR "Disease-a-month : DM"|journal! OR "Endocrinology"|journal! OR "Gastroenterology"|journal! OR "Gut"|journal! OR "Heart (British Cardiac Society)"|journal! OR "Heart & lung : the journal of critical care"|journal! OR "Hospital practice (1995)"|journal! OR "JAMA"|journal! OR "JAMA dermatology"|journal! OR "JAMA internal medicine"|journal! OR "JAMA neurology"|journal! OR "JAMA ophthalmology"|journal! OR "JAMA otolaryngology-- head & neck surgery"|journal! OR "JAMA pediatrics"|journal! OR "JAMA psychiatry"|journal! OR "JAMA surgery"|journal! OR "The Journal of allergy and clinical immunology"|journal! OR "The Journal of bone and joint surgery. American volume"|journal! OR "The Journal of clinical endocrinology and metabolism"|journal! OR "The Journal of clinical investigation"|journal! OR "Journal of clinical pathology"|journal! OR "The Journal of family practice"|journal! OR "Journal of immunology (Baltimore, Md : 1950)"|journal! OR "The Journal of infectious diseases"|journal! OR "The Journal of laryngology and otology"|journal! OR "The Journal of nervous and mental disease"|journal! OR "Journal of neurosurgery"|journal! OR "The Journal of nursing administration"|journal! OR "Journal of oral and maxillofacial surgery : official journal of the American Association of Oral and Maxillofacial Surgeons"|journal! OR "The Journal of pediatrics"|journal! OR "Journal of the Academy of Nutrition and Dietetics"|journal! OR "Journal of the American College of Cardiology"|journal! OR "Journal of the American College of Surgeons"|journal! OR "The Journal of thoracic and cardiovascular surgery"|journal! OR "The Journal of trauma and acute care surgery"|journal! OR "The Journal of urology"|journal! OR "The journals of gerontology. Series A, Biological sciences and medical sciences"|journal! OR "The journals of gerontology. Series B, Psychological sciences and social sciences"|journal! OR "Lancet (London, England)"|journal! OR "Mayo Clinic proceedings."|journal! OR "The Medical clinics of North America"|journal! OR "The Medical letter on drugs and therapeutics"|journal! OR "Medicine"|journal! OR "Neurology"|journal! OR "The New England journal of medicine"|journal! OR "The Nursing clinics of North America"|journal! OR "Nursing outlook"|journal! OR "Nursing research"|journal! OR "Obstetrics and gynecology"|journal! OR "The Orthopedic clinics of North America"|journal! OR "Pediatric clinics of North America"|journal! OR "Pediatrics"|journal! OR "Physical therapy"|journal! OR "Plastic and reconstructive surgery"|journal! OR "Postgraduate medicine"|journal! OR "Progress in cardiovascular diseases"|journal! OR "Public health reports (Washington, D.C. : 1974)"|journal! OR "Radiologic clinics of North America"|journal! OR "Radiology"|journal! OR "Rheumatology (Oxford, England)"|journal! OR "Southern medical journal"|journal! OR "Surgery"|journal! OR "The Surgical clinics of North America"|journal! OR "Translational research : the journal of laboratory and clinical medicine"|journal! OR "The Urologic clinics of North America"|journal! OR "Geriatrics"|journal! OR "The Western journal of medicine"|journal! OR "Hospitals & health networks"|journal!'
term2 = ccj+' AND 1997|pdat!:2999|pdat! AND Randomized Controlled Trial|publication type!'
#%%
Entrez.email = " @@@ " # Always tell NCBI who you are
handle = Entrez.esearch(db="pubmed", term=term2, retmax=1000000)
record = Entrez.read(handle)
handle.close()
idlist = record|"IdList"!
print(len(idlist))
length = math.ceil(len(idlist)/10000)
au = |!
au2 = |!
titles = |!
pmids = |!
years = |!
#%%
hajime = time.time()
for i in range(length):
if i < length:
ids = idlist|i*10000:(i+1)*10000!
handle = Entrez.efetch(db="pubmed", id=ids, rettype="medline", retmode="text")
records = Medline.parse(handle)
for record in records:
if "AU" in record:
authornumber1 = |len(record|"AU"!)!
else:
authornumber1 = |0!
au = au + authornumber1
if "IR" in record:
authornumber2 = |len(record|"IR"!)!
else:
authornumber2 = |0!
au2 = au2 + authornumber2
title = |record|"TA"!!
titles = titles + title
pmid = |record|"PMID"!!
pmids = pmids + pmid
if "DP" in record:
year = |record|"DP"!!
else:
year = |'NaN'!
years = years + year
else:
ids = idlist|i*10000:len(idlist)!
handle = Entrez.efetch(db="pubmed", id=ids, rettype="medline", retmode="text")
records = Medline.parse(handle)
for record in records:
if "AU" in record:
authornumber1 = |len(record|"AU"!)!
else:
authornumber1 = |0!
au = au + authornumber1
if "IR" in record:
authornumber2 = |len(record|"IR"!)!
else:
authornumber2 = |0!
au2 = au2 + authornumber2
title = |record|"TA"!!
titles = titles + title
pmid = |record|"PMID"!!
pmids = pmids + pmid
if "DP" in record:
year = |record|"DP"!!
else:
year = |'NaN'!
years = years + year
df = pd.DataFrame(data = {'pmid':pmids,'authornumber1':au,'investigater':au2,'title':titles,'year':years})
owari = time.time()
elapsed_time = owari - hajime
print(f"elapsed_time: {elapsed_time}")
#%%
df|"years"! = df|'year'!.str|:4!
df|'authors'! = df|'authornumber1'!+ df|'investigater'!
#ccj all articles
df.to_excel("ccj_all_articles_since_1997.xlsx",index=False)
#%%post-processing
authormedian = pd.pivot_table(df, index='years',
aggfunc=np.median)
authormedian = authormedian|'authors'!
df|'ronbun'! = 1
df2 = df.loc|:,|'ronbun','years'!!
ronbunsu = pd.pivot_table(df2, index='years',
aggfunc=np.sum)
ccjshukei = pd.concat(|ronbunsu, authormedian!,axis=1)
ccjshukei.to_excel("summarize_ccj_all_articles_since_1997.xlsx")
#%% all
Entrez.email = "@@@" # Always tell NCBI who you are
handle = Entrez.esearch(db="pubmed", term=term, retmax=100000000)
record = Entrez.read(handle)
handle.close()
idlist = record|"IdList"!
print(len(idlist))
length = math.ceil(len(idlist)/10000)
au = |!
au2 = |!
titles = |!
pmids = |!
dps = |!
edats = |!
#%%
hajime = time.time()
for i in range(length):
if i < length:
ids = idlist|i*10000:(i+1)*10000!
handle = Entrez.efetch(db="pubmed", id=ids, rettype="medline", retmode="text")
records = Medline.parse(handle)
for record in records:
title = |record|"TA"!!
titles = titles + title
pmid = |record|"PMID"!!
pmids = pmids + pmid
edat = |record|"EDAT"!!
edats = edats + edat
if "DP" in record:
dp = |record|"DP"!!
else:
dp = |'NaN'!
dps = dps + dp
else:
ids = idlist|i*10000:len(idlist)!
handle = Entrez.efetch(db="pubmed", id=ids, rettype="medline", retmode="text")
records = Medline.parse(handle)
for record in records:
title = |record|"TA"!!
titles = titles + title
pmid = |record|"PMID"!!
pmids = pmids + pmid
edat = |record|"EDAT"!!
edats = edats + edat
if "DP" in record:
dp = |record|"DP"!!
else:
dp = |'NaN'!
dps = dps + dp
print(i)
df = pd.DataFrame(data = {'pmid':pmids,'title':titles,'dps':dps,'edats':edats})
owari = time.time()
elapsed_time = owari - hajime
print(f"elapsed_time: {elapsed_time}")
#%%
df|"dps_years"! = df|'dps'!.str|:4!
df|"edats_years"! = df|'edats'!.str|:4!
df2 = df.loc|:,|'title',"dps_years","edats_years"!!
df.to_excel("MEDLINE_all_articles_since_1997.xlsx",index=False)
#%%
kara = |!
for i in range(24):
i = i+1997
i = str(i)
df3 = df2|df2|'years'!==i!
count = len(df3|'title'!.value_counts())
kara = kara+|count!
zassisu= pd.Series(kara)
zassisu.to_excel("number_of_journals_in_MEDLINE.xlsx",index=False)
#%%
nen1 = df2|"edats_years"!.value_counts()
nen2 = df2|"dps_years"!.value_counts()
nen1 = nen1.sort_index()
nen2 = nen2.sort_index()
#%%
nen = pd.concat(|nen1,nen2!,axis=1)
nen.to_excel('number_of_articles_in_MEDLINE_in_each_year.xlsx')
